# Effects of Structured Supervised Exercise Training or Motivational Counseling on Pregnant Women’s Physical Activity Level: FitMum - Randomized Controlled Trial

**DOI:** 10.2196/37699

**Published:** 2022-07-20

**Authors:** Signe de Place Knudsen, Saud Abdulaziz Alomairah, Caroline Borup Roland, Anne Dsane Jessen, Ida-Marie Hergel, Tine D Clausen, Jakob Eg Larsen, Gerrit van Hall, Andreas Kryger Jensen, Stig Molsted, Jane M Bendix, Ellen Løkkegaard, Bente Stallknecht

**Affiliations:** 1 Department of Gynecology and Obstetrics Copenhagen University Hospital—North Zealand Hillerod Denmark; 2 Department of Biomedical Sciences University of Copenhagen Copenhagen Denmark; 3 Public Health Department College of Health Sciences Saudi Electronic University Riyadh Saudi Arabia; 4 Department of Clinical Medicine University of Copenhagen Copenhagen Denmark; 5 Department of Applied Mathematics and Computer Science Technical University of Denmark Kgs. Lyngby Denmark; 6 Clinical Biochemistry Clinical Metabolomics Core Facility Rigshospitalet Copenhagen Denmark; 7 Section of Biostatistics Department of Public Health University of Copenhagen Copenhagen Denmark; 8 Department of Clinical Research Copenhagen University Hospital—North Zealand Hillerod Denmark

**Keywords:** motivation, physical activity, pregnancy, pregnant, RCT, randomized controlled trial, intervention, commercial activity tracker, tracker, COVID-19, maternal health, doubly labeled water, physical activity questionnaire, women's health, maternal, maternity, digital health, exercise, fitness, health outcome

## Abstract

**Background:**

Physical activity (PA) during pregnancy is an effective and safe way to improve maternal health in uncomplicated pregnancies. However, compliance with PA recommendations remains low among pregnant women.

**Objective:**

The purpose of this study was to evaluate the effects of offering structured supervised exercise training (EXE) or motivational counseling on PA (MOT) during pregnancy on moderate-to-vigorous intensity physical activity (MVPA) level. Additionally, complementary measures of PA using the Pregnancy Physical Activity Questionnaire (PPAQ) and gold standard doubly labeled water (DLW) technique were investigated. The hypotheses were that both EXE and MOT would increase MVPA in pregnancy compared with standard care (CON) and that EXE would be more effective than MOT. In addition, the association between MVPA and the number of sessions attended was explored.

**Methods:**

A randomized controlled trial included 220 healthy, inactive pregnant women with a median gestational age of 12.9 (IQR 9.4-13.9) weeks. A total of 219 women were randomized to CON (45/219), EXE (87/219), or MOT (87/219). The primary outcome was MVPA (minutes per week) from randomization to the 29th gestational week obtained by a wrist-worn commercial activity tracker (Vivosport, Garmin International). PA was measured by the activity tracker throughout pregnancy, PPAQ, and DLW. The primary outcome analysis was performed as an analysis of covariance model adjusting for baseline PA.

**Results:**

The average MVPA (minutes per week) from randomization to the 29th gestational week was 33 (95% CI 18 to 47) in CON, 50 (95% CI 39 to 60) in EXE, and 40 (95% CI 30 to 51) in MOT. When adjusted for baseline MVPA, participants in EXE performed 20 (95% CI 4 to 36) minutes per week more MVPA than participants in CON (*P*=.02). MOT was not more effective than CON; EXE and MOT also did not differ. MVPA was positively associated with the number of exercise sessions attended in EXE from randomization to delivery (*P*=.04). Attendance was higher for online (due to COVID-19 restrictions) compared with physical exercise training (*P*=.03). Adverse events and serious adverse events did not differ between groups.

**Conclusions:**

Offering EXE was more effective than CON to increase MVPA among pregnant women, whereas offering MOT was not. MVPA in the intervention groups did not reach the recommended level in pregnancy. Changing the intervention to online due to COVID-19 restrictions did not affect MVPA level but increased exercise participation.

**Trial Registration:**

ClinicalTrials.gov NCT03679130; https://clinicaltrials.gov/ct2/show/NCT03679130

**International Registered Report Identifier (IRRID):**

RR2-10.1136/bmjopen-2020-043671

## Introduction

Physical activity (PA) is a safe and effective way to improve maternal health in uncomplicated pregnancies [1,2]. Regular PA during pregnancy reduces the risk of gestational weight gain, gestational diabetes mellitus, gestational hypertension, preeclampsia, cesarean delivery [3], and depression [4]. In addition, lifestyle interventions during pregnancy may improve offspring health by improving placental function [5,6], reducing the risk of preterm delivery [3], and normalizing birth weight [7,8]. Nevertheless, compliance with PA recommendations remains low among pregnant women worldwide [9]. Therefore, a pressing issue to address is how to implement PA in the everyday life of pregnant women.

A diverse range of approaches to PA interventions exists, of which structured supervised exercise training and motivational counseling on PA are used widely in the literature [10]. Supervised exercise training with scheduled exercise sessions provides a standard approach to increase PA in pregnant women. Recognizing the needs of an individually tailored approach [11,12], motivational counseling focuses on PA behavior has also been shown to reduce the decline or even increase PA during pregnancy [13-15]. Structured supervised exercise and motivational counseling on PA have been applied separately in studies of pregnant women [16-26], but a direct comparison of the two approaches to increase PA during pregnancy has not yet been performed.

The primary objective of FitMum was to evaluate the effects of offering structured supervised exercise training (EXE) or motivational counseling on PA (MOT) compared to standard care (CON) on moderate-to-vigorous intensity PA (MVPA) in pregnant women as determined by a wrist-worn commercial activity tracker. Secondary measures of PA were obtained by the Danish version of the Pregnancy Physical Activity Questionnaire (PPAQ-DK) [27,28] and by the gold standard doubly labeled water (DLW) technique [29-31]. The hypotheses were that both EXE and MOT would increase MVPA in pregnancy compared to CON and that EXE would be more effective than MOT [32,33]. In addition, the association between MVPA and the number of sessions attended was explored.

## Methods

### Ethics Approval

The study was approved by the Danish National Committee on Health Research Ethics (H-18011067) and the Danish Data Protection Agency (P-2019-512) and registered at ClinicalTrials.gov (NCT03679130). The study adheres to the principles of the Helsinki declaration. Written informed consent was obtained at inclusion.

### Patient and Public Involvement

The development of FitMum was inspired by stakeholders: 27 semistructured interviews with Danish pregnant women, midwives, and obstetricians were performed to explore the feasibility, facilitators, and barriers to PA during pregnancy.

### Participants and Trial Design

**Figure 1:**
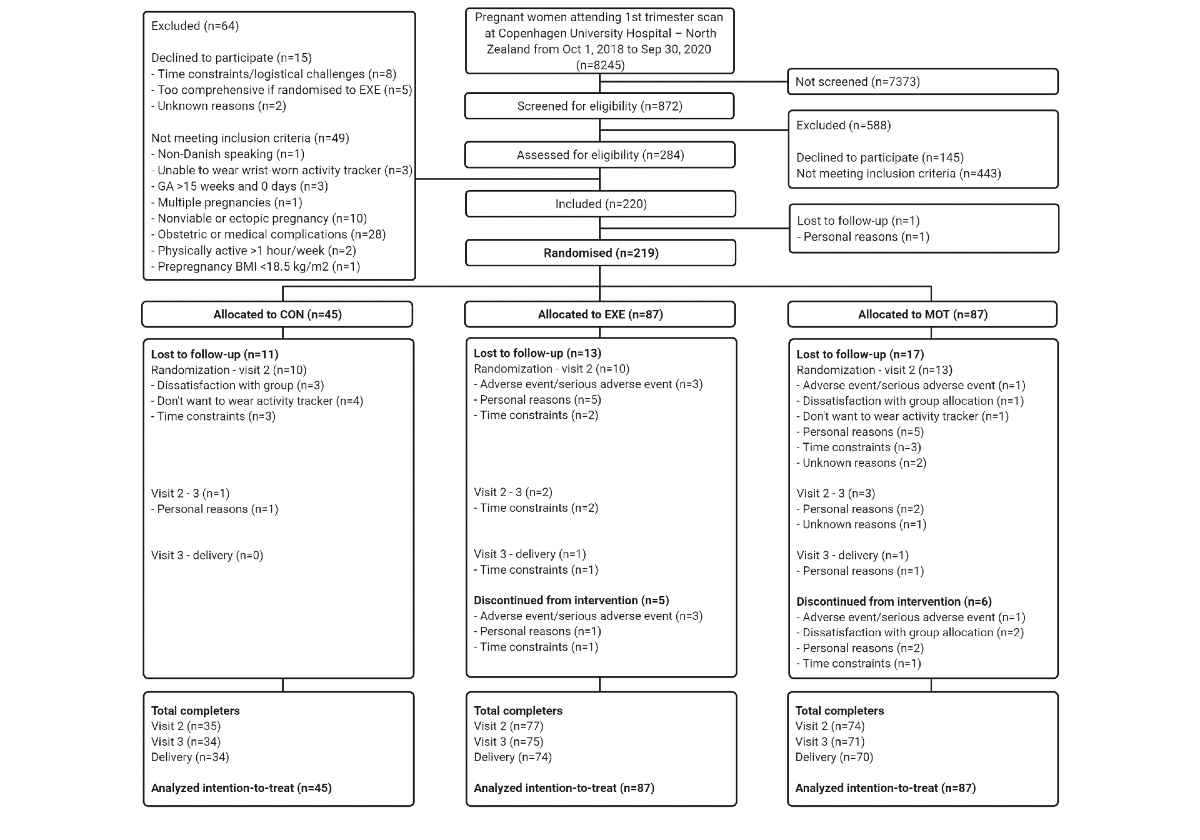
Flowchart of the FitMum randomized controlled trial including enrollment, study group allocation, follow-up, and data analysis. GA: gestational age; CON: standard care; EXE: structured supervised exercise training; MOT: motivational counseling on physical activity.

FitMum was a single-site randomized controlled trial (RCT) conducted from 2018-2021 at the Department of Gynecology and Obstetrics at Copenhagen University Hospital–North Zealand, Denmark [32]. A total of 220 healthy, inactive pregnant women with gestational ages of ≤15 weeks and 0 days were included (visit 1). Participants were randomized 1:2:2 into CON, EXE, and MOT groups, respectively (Figure 1). Participants were invited to a test visit at the 29th gestational week (visit 2) and the 35th gestational week (visit 3).

### Interventions

All 3 groups were offered standard maternal care. The EXE group was offered 1-hour group-based supervised exercise training at moderate intensity 3 times per week in a gym and swimming pool. The MOT group was offered 4 individual and 3 group PA motivational counseling face-to-face sessions of 1 to 2 hours duration during pregnancy and 1 weekly, personalized text message to support PA. The motivation technique applied is inspired by motivational interviewing [34], self-determination theory [35], and behavior change techniques [36].

Interventions ran from randomization until delivery. The target PA level for the EXE and MOT groups was at least 30 minutes per day at a moderate intensity as recommended in Denmark to healthy pregnant women [37]. Interventions were converted into online versions during the COVID-19 pandemic restrictions introduced in Denmark on March 11, 2020, and throughout the study period. The EXE group could access the swimming pool for 3 months during this period.

### Outcome Measures

**Figure 2:**
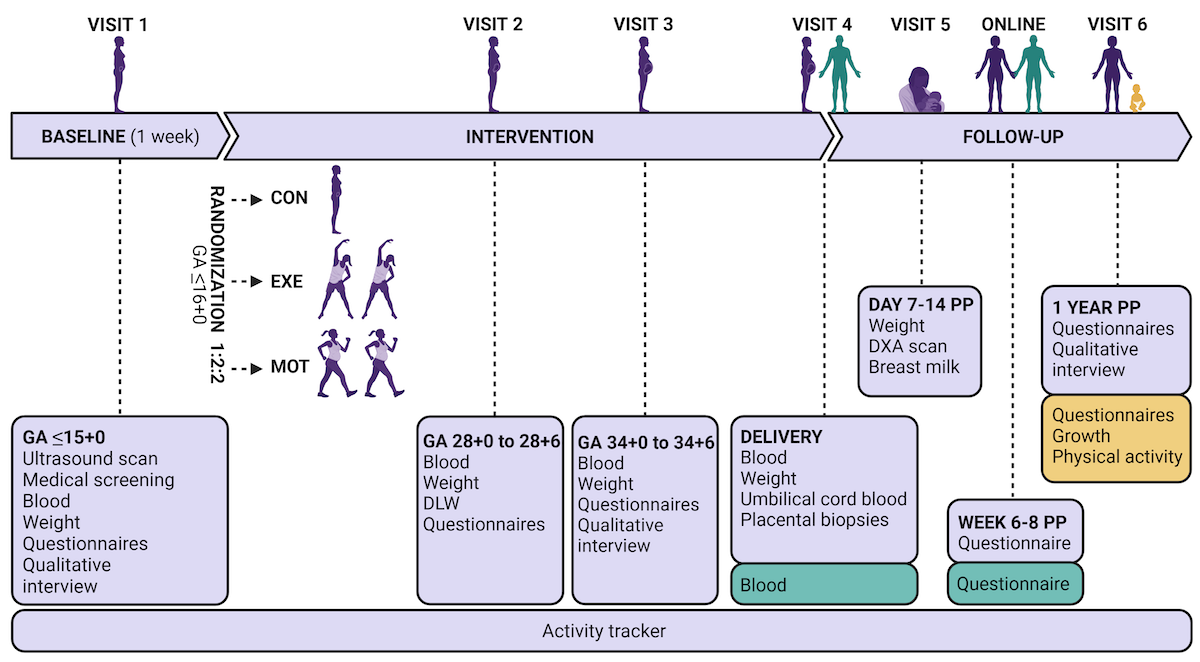
Schedule of visits. GA: gestational age; CON: standard care; EXE: structured supervised exercise training; MOT: motivational counseling on physical activity; DLW: doubly labeled water technique; PP: postpartum; DXA: dual-energy x-ray absorptiometry.

The data collection procedures are illustrated in Figure 2. PA was continuously monitored by the activity tracker from randomization to delivery, by PPAQ at visits 1, 2, and 3, and by DLW at visit 2.

### Activity Tracker

The primary outcome was MVPA (minutes per week) from randomization to visit 2. PA was from inclusion to delivery continuously captured by a wrist-worn commercial activity tracker (Vivosport, Garmin International) [38] with a built-in heart rate monitor and accelerometer. Baseline PA was captured from inclusion to randomization (6 full days). PA with a metabolic equivalent of task (MET) value of ≥3 in bouts of at least 10 consecutive minutes was recorded automatically as MVPA by the activity tracker [38]. Secondary outcomes measured by the activity tracker were PA duration at moderate and vigorous intensities; steps; active time; active kilocalories; floors climbed; and minimum, maximum, resting, and average heart rate from randomization to delivery. At inclusion, the activity tracker was preset with PA notifications turned off and an identical face of the tracker showing only clock and battery level. After randomization, women in the MOT group were encouraged to personalize the tracker with, for example, individual goal settings and PA notifications as part of the intervention. Interaction with the tracker was neither encouraged nor controlled for the EXE and CON groups. Throughout the study period tracker software was automatically updated [38].

### Danish Version of the PPAQ

PA was digitally self-reported by participants using the PPAQ-DK [28] at visits 1, 2, and 3. The questionnaire assesses PA related to everyday activities during the current trimester (eg, household, occupational, sports, and transportation) [27].

### DLW Technique

Participants collected 2 baseline urine samples prior to visit 2, drank the DLW dose at the visit, and then collected and stored 5 postdose urine samples at home on days 1, 4, 7, 11, and 14 and later at −80°C. [31,39]. The calculation of total energy expenditure (TEE) was based on the Weir equation [39], and the active energy expenditure (AEE) was calculated by subtracting the basal metabolic rate (BMR) from the TEE. BMR was estimated by an equation appropriate for pregnant women [40]. PA level (PAL) was calculated by dividing TEE by BMR.

### Activity Tracker Data Management

PA was transferred via Bluetooth from the activity tracker to the Garmin Connect app (Garmin International) [38] from which Fitabase (Small Steps Labs LLC) obtained the data via the programming interface. PA was monitored through Fitabase, and participants were reminded if the activity trackers were not synchronizing. PA data were downloaded from Fitabase, processed, and cleaned in R software (R Foundation for Statistical Computing).

### Statistical Analyses

Statistical analyses were performed according to the statistical analysis plan, which includes a sample size calculation [33] using R. Data are presented as means and standard deviations for symmetric distributions, medians and IQRs for skewed data, and frequencies and percentages for categorical variables. The level of statistical significance was 5% except for the primary hypothesis which consisted of 2 subhypotheses; the type I error for each hypothesis test was a priori set to 2.5% to obtain a family-wise error rate of 5%. Wald-based 95% CI were given for all reported estimates [33]. Intention-to-treat analyses using all randomized participants were performed for the primary outcome. Missing observations in tracker data due to nonwear time were imputed by multiple imputations in 25 data sets using a prespecified seed, preselected baseline variables (body weight, age, PA, educational level, and parity), and the random forest imputation model from the mice R package [41]. A statistician blinded for the intervention performed the imputation and the primary outcome analysis as an analysis of covariance model adjusting for baseline PA. MVPA before and during the COVID-19 pandemic was compared within groups with a linear regression model. Cumulative trajectories were estimated from the imputed data using a generalized additive model with a penalized regression spline with point-wise 95% confidence bands estimated by a bootstrap procedure [42]. For the PPAQ-DK outcome, a constrained linear mixed model was fitted with the observation times as a factor [43]. Both within and between-group effects were reported as estimated differences in means. For the DLW outcome, a one-way analysis of variance was used to compare the 3 group averages. For the DLW outcome, a 1-way analysis of variance was used to compare the 3 group averages. Linear regression was used to model the relationship between attended intervention sessions and attained MVPA in the EXE and MOT groups.

## Results

### Participants and Adherence to Interventions

In total, 220 pregnant women were included from October 2018 to October 2020. Of those, 219 were randomly allocated to CON (45/219), EXE (87/219) or MOT (87/219; Figure 1). Maternal baseline characteristics are presented in Table 1.

From randomization to visit 2, 15.1% (33/219) of participants were lost to follow-up (CON: 10/45, 22%; EXE: 10/87, 11%; MOT: 13/87, 15%). The main reason (18/33, 55%) was personal matters (eg, time consumed with participation or family reasons). From randomization to delivery, 18.7% (41/219) of participants were lost to follow-up, and proportions were similar across groups (Figure 1).

Participants randomized to EXE participated in 1.4 (95% CI 1.2 to 1.6) exercise sessions per week from randomization to visit 2, and 1.3 (95% CI 1.1 to 1.5) exercise sessions per week from randomization to delivery. Participants randomized to the MOT group joined 5.2 (95% CI 4.7 to 5.7) counseling sessions during their pregnancy.

**Table 1:** Baseline characteristics of randomized participants.

Characteristics			All (n=219)		CON^a^ (n=45)		EXE^b^ (n=87)		MOT^c^ (n=87)
Age (years), mean (SD)			31.5 (4.3)		32.0 (4.6)		31.1 (4.3)		31.7 (4.1)
Gestational age at inclusion (weeks), median (IQR)			12.9 (9.4-13.9)		12.9 (9.7-13.9)		12.6 (9.3-13.7)		12.9 (9.6-13.9)
Weight (kg), mean (SD)			75.4 (15.3)		72.0 (13.7)		76.2 (17.4)		76.3 (13.8)
Prepregnancy BMI^d^ (kg/m^2^), median (IQR)			24.1 (21.8-28.7)		23.5 (21.3-26.8)		25.2 (21.6-29.8)		24.1 (22.4-28.9)
Nulliparity, n (%)			82 (37.4)		16 (3.56)		40 (46.0)		26 (29.9)
**Educational level, n (%)**									
	School ≥12 years	191 (87.2)		41 (91.1)		74 (85.1)		76 (87.4)	
	Further education ≥3 years	175 (79.9)		33 (73.3)		73 (83.9)		69 (79.3)	
	Employed/studying	199 (90.9)		39 (86.7)		83 (95.4)		77 (88.5)	

^a^CON: standard care.

^b^EXE: structured supervised exercise training.

^c^MOT: motivational counseling on physical activity.

^d^Prepregnancy BMI is calculated based on n=218 (CON: 45/218, EXE: 86/218, MOT: 87/218) due to a missing value.

### PA by Activity Tracker

#### Moderate-to-Vigorous Intensity Physical Activity

**Figure 3:**
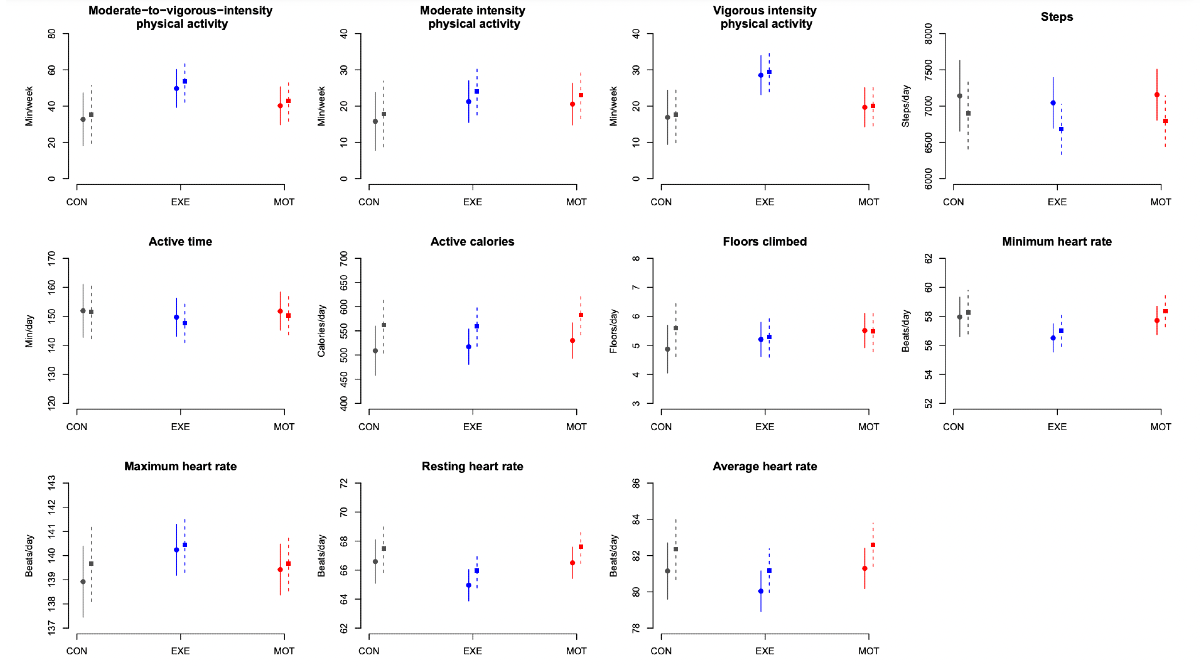
Moderate-to-vigorous intensity physical activity (primary outcome) and additional activity tracker outcomes (mean and 95% CI) from randomization to visit 2 (29th week of gestation; solid line) and from randomization to delivery (dotted line). MVPA: moderate-to-vigorous intensity physical activity; CON: standard care; EXE: structured supervised exercise training; MOT: motivational counseling on physical activity.

The average MVPA (minutes per week) from randomization to visit 2 was 33 (95% CI 18 to 47) in CON, 50 (95% CI 39 to 60) in EXE, and 40 (95% CI 30 to 51) in MOT (Figure 3). When adjusted for baseline MVPA, participants in EXE performed 20 (95% CI 4 to 36) minutes per week more MVPA than participants in CON (*P*=.02; Multimedia Appendix 1).

The same pattern was seen throughout the entire pregnancy, hence the unadjusted average MVPA (minutes per week) was 35 (95% CI 19 to 51) in CON, 54 (95% CI 42 to 65) in EXE and 43 (95% CI 32 to 55) in MOT from randomization to delivery (Figure 3). Throughout pregnancy, participants in EXE performed 21 (95% CI 3 to 39) minutes per week more MVPA than participants in CON when adjusted for baseline MVPA (*P*=.02; Multimedia Appendix 1).

There were no significant differences in adjusted MVPA between CON and MOT (randomization to visit 2: *P*=.23; randomization to delivery: *P*=.27) or between MOT and EXE (randomization to visit 2: *P*=.14; randomization to delivery: *P*=.15; Multimedia Appendix 1).

**Figure 4:**
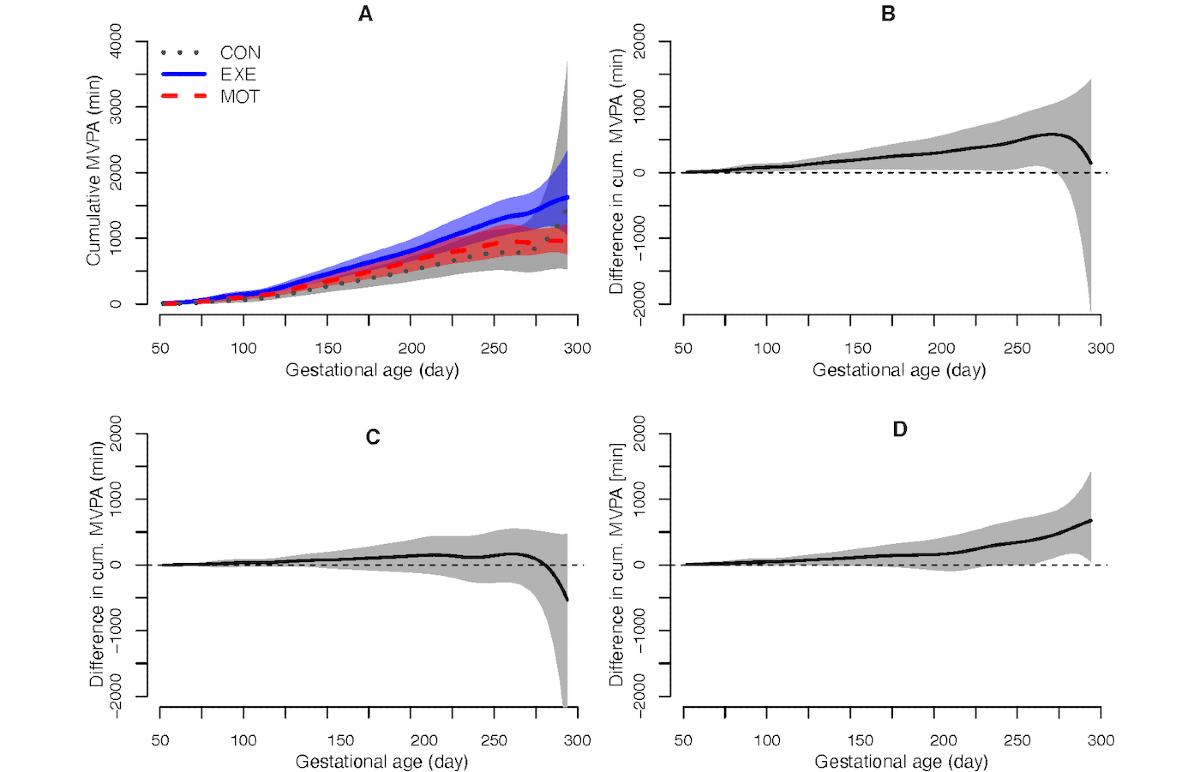
Cumulative moderate-to-vigorous intensity physical activity from randomization to delivery: (A) group averages, (B) EXE vs CON, (C) MOT vs CON, and (D) EXE vs MOT. MVPA: moderate-to-vigorous intensity physical activity; CON: standard care; EXE: structured supervised exercise training; MOT: motivational counseling on physical activity.

**Figure 5:**
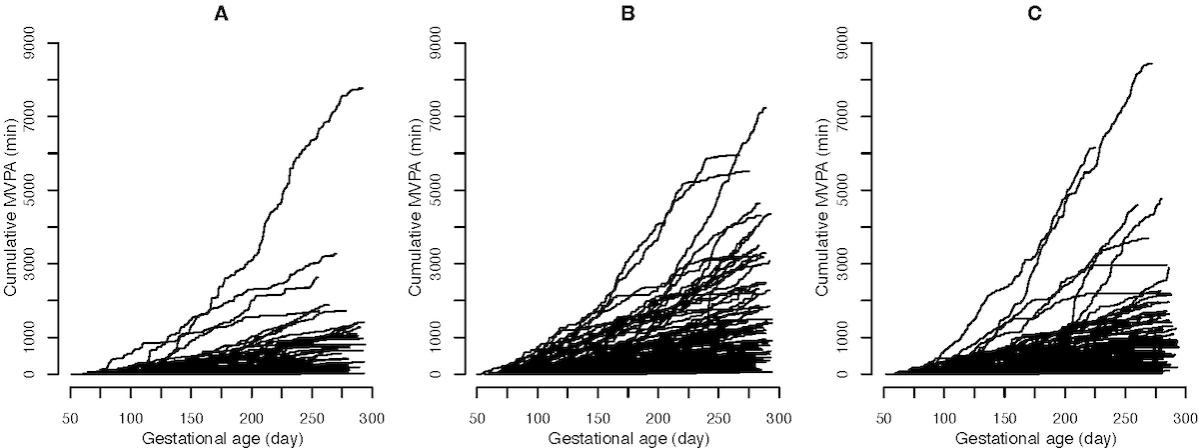
Individual cumulative moderate-to-vigorous intensity physical activity from randomization to delivery in (A) standard care, (B) structured supervised exercise training, and (C) motivational counseling on physical activity. MVPA: moderate-to-vigorous intensity physical activity.

Unplanned analysis on cumulative MVPA from randomization to delivery revealed great variability and that EXE tended to have more MVPA compared with MOT, which became significant in the late part of pregnancy (Figures 4 and 5). The same tendency was seen between CON and EXE, but the difference was insignificant. Cumulative MVPA did not differ between CON and MOT.

The number of training sessions attended in EXE from randomization to delivery was positively associated with MVPA level (*P*=.04). No association was present between the number of sessions attended in MOT and MVPA (*P*=.14).

#### COVID-19 Sensitivity Analysis

MVPA (minutes per week) did not differ between participants included before the COVID-19 pandemic (physical intervention only, 120/219) and during the COVID-19 pandemic (online intervention only, 63/219) in either CON (–14, 95% CI –49 to 22; *P*=.44), EXE (–16, 95% CI –42 to 11; *P*=.25), or MOT (–6, 95% CI –37 to 25; *P*=.712; Multimedia Appendix 2).

Women in EXE offered the online intervention only participated in more exercise sessions per week than women offered the physical intervention only (online: 1.6, 95% CI 1.3 to 2.0 and physical: 1.1, 95% CI 0.9 to 1.4; *P*=.03). Participants in EXE attended on average 4.9 swimming pool sessions during the online intervention period. The number of MOT sessions attended did not differ between women who were offered the intervention before or during the COVID-19 pandemic (physical: 5.3, 95% CI 4.6 to 6.0 and online: 5.6, 95% CI 4.8 to 6.4; *P*=.97). Participants included before the COVID-19 pandemic and delivered during (36/219) were excluded in this analysis based on their mixed intervention.

### Secondary Activity Tracker Outcomes

All tracker outcomes are presented in Figure 3 and accompanying statistics in Multimedia Appendix 1. PA at a vigorous intensity (minutes per week) was higher in EXE than in both CON and MOT (CON vs EXE: randomization to visit 2: 13, 95% CI 4 to 22; randomization to delivery: 13, 95% CI 4 to 22; MOT vs EXE: randomization to visit 2: 9, 95% CI 1 to 16, randomization to delivery: 9, 95% CI 1 to 17). In addition, the maximum heart rate was 2 (95% CI 0.3 to 3) beats per minute higher in EXE compared with CON from randomization to visit 2. No other tracker outcomes differed between groups.

### PA by PPAQ-DK

**Figure 6:**
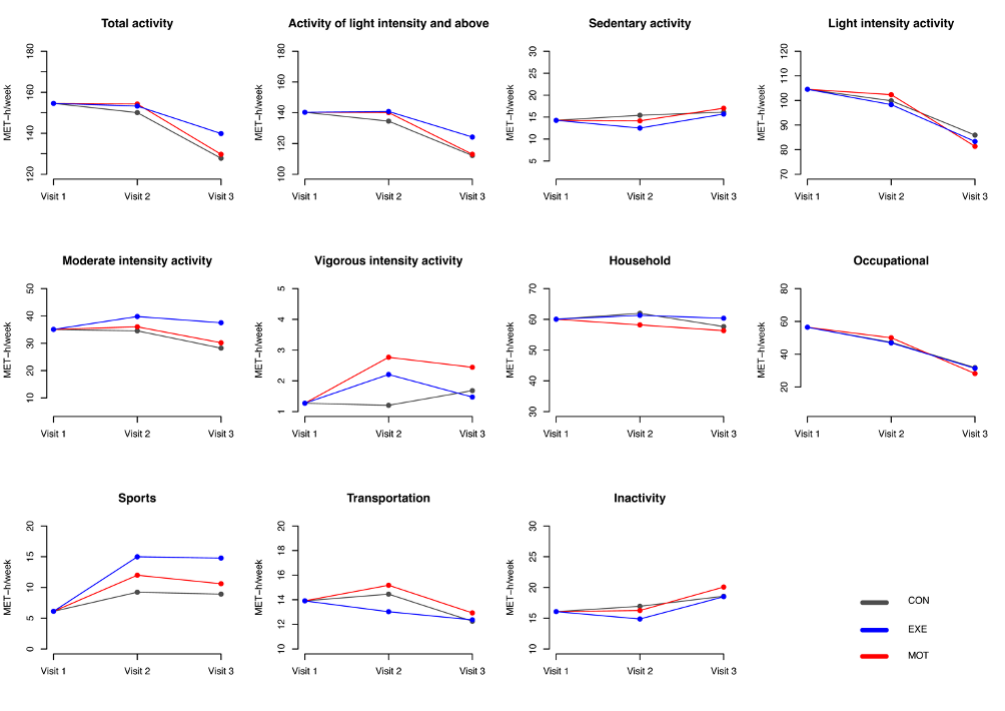
Baseline-constrained comparison between groups based on the means of physical activity level from the Danish version of the Pregnancy Physical Activity Questionnaire. MET: metabolic equivalent of task; CON: standard care; EXE: structured supervised exercise training; MOT: motivational counseling on physical activity.

PPAQ-DK was completed for visits 1, 2, and 3 by 100% (219/219), 83.1% (182/219), and 77.2% (169/219) participants, respectively. Figure 6 shows the PA behaviors categorized by intensity and type. Differences between and within groups are shown in Multimedia Appendix 3 and Multimedia Appendix 4.

Total activity did not change from visit 1 to visit 2 in CON, EXE, or MOT, but PA decreased significantly from visit 1 to visit 3 in all groups (Multimedia Appendix 4). PA at moderate intensity was maintained at the same level over the course of pregnancy in CON, EXE, and MOT. However, participants in MOT increased PA at vigorous intensity from visit 1 to visit 2 and visit 1 to visit 3 (Multimedia Appendix 4). When combined (MVPA), the activity level (MET hours per week) did not change through pregnancy in any of the groups (CON: visit 1-2: –1, *P*=.90; visit 1-3: –4, *P*=.36; EXE: visit 1-2: 4, *P*=.10; visit 1-3: 1, *P*=.61; MOT: visit 1-2: 2, *P*=.40; visit 1-3: –5, *P*=.37; data not shown).

The MET hours per week spent at sports activities increased significantly from visit 1 to visit 2 and visit 1 to visit 3 for both EXE and MOT, while no changes were observed in CON (Multimedia Appendix 4). A comparison between groups revealed that sports was significantly higher in EXE compared with CON and MOT at both visit 2 and visit 3 (Multimedia Appendix 3).

### PA by DLW

A total of 134 participants (CON: 24/45, EXE: 53/87, MOT: 57/87) completed the DLW test and were included in the analysis. TEE (*P*=.14), AEE (*P*=.38), and PAL (*P*=.66) did not differ between groups (TEE [kcal per day]: CON 2215 [SD 238], EXE 2330 [SD 264], MOT 2331 [SD 260]; AEE [kcal per day]: CON 543 [SD 106], EXE 592 [SD 160], MOT 587 [SD 155]; and PAL [TEE/BMR]: CON 1.33 [SD 0.06], EXE 1.35 [SD 0.11], MOT 1.34 [SD 0.09]; Multimedia Appendix 5).

### Adverse Events and Serious Adverse Events

Adverse events and serious adverse events from inclusion to delivery among all participants did not differ between groups (Multimedia Appendices 6-8).

## Discussion

### Principal Findings

FitMum aimed to investigate the effects of offering EXE or MOT to generate evidence about how to implement PA in healthy pregnant women’s lives. We hypothesized that both EXE and MOT would increase MVPA in pregnancy compared with CON but that EXE would be more effective than MOT [33]. The study confirmed that EXE was more effective than CON, whereas MOT was not more effective than CON, and EXE and MOT did not differ. The number of adverse events and serious adverse events did not differ between groups.

### Effectiveness of PA Interventions On PA Level In Pregnant Women

Several previous RCTs have used strategies like ours to examine how to increase PA in pregnant women and at the same time assessed the PA level by objective methods [13,24,26,44,45]. Seneviratne et al [24] conducted a 16-week stationary biking program in overweight and obese pregnant women and reported improved aerobic fitness compared to controls. When determining PA objectively by accelerometry, Hayman et al [26] found an immediate increase in MVPA after 4 weeks of tailored PA advice and access to a resource library. On the contrary, no increase in PA as determined by accelerometry was found after a combined aerobic and strength exercise program [44], face-to-face individual PA consultations [13], or app-based PA behavior change techniques [45].

Women in EXE were encouraged to participate in 3 hours of EXE per week, but the participants attended on average less than half of the sessions, and throughout their pregnancy, the MVPA level was only a third (54 of 150 minutes per week) of the internationally recommended amount [2]. As expected, MVPA was positively associated with the number of exercise sessions attended. Noticeably, EXE had a higher level of vigorous intensity PA compared with both CON and MOT. This was supported by a higher maximum heart rate among EXE. Exercising at vigorous intensity is in accordance with recent suggestions for healthy pregnant women [46,47]. MOT had a high intervention attendance, but even though MOT contained face-to-face counseling, text messaging, activity tracker use, and behavior change techniques as recommended [13,48,49], we found no effect on MVPA compared with CON. The processes behind this finding are currently being assessed via mixed methods. The cumulative MVPA in EXE was significantly higher compared with MOT in the late part of pregnancy, and the same tendency was seen between CON and EXE. Interestingly, women who received the online EXE intervention due to COVID-19 restrictions joined 45% more exercise sessions compared with those who received the physical intervention.

### Methodologies Used to Determine PA

Combining 3 different methodologies to assess PA using objective (activity tracker and DLW) and subjective (PPAQ-DK) methods provides insight into the complexity of PA. The activity tracker offers 24/7 measures of PA, and due to its convenience the tracker can be worn for a long period of time. However, commercial trackers are not designed for research purposes, and tracker algorithms are unknown. The PPAQ is considered one of the most valid and reliable questionnaires for the assessment of PA in pregnant women [27,50], but the inherent bias of self-reported PA is inevitable. The administration of the PPAQ-DK may have led to a heightened awareness of activity among participants [50], especially for members of the MOT group, who received a thorough review of their PA level at the counseling sessions. This might explain the perceived increase in vigorous intensity PA in MOT as determined by PPAQ-DK. DLW is the reference method for the determination of free-living energy expenditure and has previously been used to estimate PA level in pregnant women [39,51], but this is the first intervention study in pregnant women to include DLW. We found no significant differences between groups in TEE, AEE, or PAL, but this might be due to a lack of power, as TEE and AEE were 50 to 100 kcal per day higher in EXE and MOT compared with CON. On the other hand, active kilocalories recorded by the tracker and total activity obtained from the PPAQ-DK, which are equivalent to AEE from DLW, did not differ between groups. Therefore, the total activity probably did not differ between groups.

### Strengths and Limitations

FitMum is the first RCT to compare the effectiveness of 2 different PA interventions in pregnant women. Strengths comprise the robust design based on the power of randomization, which leaves the internal validity high, and the comprehensive assessment of PA. The primary outcome was measured by a commercial activity tracker, which measured PA continuously, but no data on the validity of the tracker activity measurements has been published. The activity tracker may increase PA due to its motivational impact [49,52], but it might also not capture all activities. Notably, by default the tracker only reported activities with a MET value of ≥3 in bouts of at least 10 consecutive minutes as MVPA [38], and this might partly explain the relatively low MVPA in this study. An additional limitation was the impact of COVID-19 and the need to convert the physical interventions into online ones.

### Conclusions

Findings from this RCT demonstrate that offering EXE is more effective than CON to implement MVPA in healthy pregnant women’s lives. Offering MOT was not more effective than CON; EXE and MOT also did not differ. The MVPA in the intervention groups did not reach the recommended PA level in pregnancy. Changing the intervention to online due to COVID-19 restrictions did not affect MVPA level but increased exercise participation. Based on the most effective intervention on MVPA during pregnancy (EXE) and the increased level of EXE sessions attended in the online setup during the COVID-19 pandemic, it might be beneficial to add home-based, online exercise sessions in future prenatal PA interventions.

## Appendix

Multimedia Appendix 1 Comparison between groups based on imputed activity tracker datasets (intention-to-treat analysis) from randomization to visit 2 and delivery, respectively.

Multimedia Appendix 2 Moderate-to-vigorous intensity physical activity before and during the COVID-19 pandemic.

Multimedia Appendix 3 Pregnancy Physical Activity Questionnaire outcome differences.

Multimedia Appendix 4 Pregnancy Physical Activity Questionnaire outcome descriptive statistics.

Multimedia Appendix 5 One-way analysis of variance test of the doubly labeled water outcomes.

Multimedia Appendix 6 Summary of adverse events and serious adverse events.

Multimedia Appendix 7 All adverse events.

Multimedia Appendix 8 All serious adverse events.

## Abbreviations

AEE: active energy expenditure
BMR: basal metabolic rate
CON: standard care
DLW: doubly labeled water
EXE: structured supervised exercise training
MET: metabolic equivalent of task
MOT: motivational counseling
MVPA: moderate-to-vigorous intensity physical activity
PA: physical activity
PAL: physical activity level
PPAQ-DK: Danish Pregnancy Physical Activity Questionnaire
RCT: randomized controlled trial
TEE: total energy expenditure
