# Supplementary material for: Effects of Structured Supervised Exercise Training or Motivational Counseling on Pregnant Women’s Physical Activity Level: FitMum - Randomized Controlled Trial
Source: J Med Internet Res. 2022 Jul 20;24(7):e37699. doi: 10.2196/37699 (PMC9350815; doi:10.2196/37699)
Supplement: Multimedia Appendix 1 [file jmir_v24i7e37699_app1.docx]

Multimedia Appendix 1. Comparison between groups based on imputed activity tracker datasets (intention-to-treat analysis) from randomization to visit 2 and delivery, respectively.

|  | CON vs EXE | | | | CON vs MOT | | | | MOT vs EXE | | | |
| --- | --- | --- | --- | --- | --- | --- | --- | --- | --- | --- | --- | --- |
|  | **Visit 2** | | **Delivery** | | **Visit 2** | | **Delivery** | | **Visit 2** | | **Delivery** | |
|  | **Differences [95% CI]** | ***P value*** | **Differences [95% CI]** | ***P value*** | **Differences [95% CI]** | ***P value*** | **Differences [95% CI]** | ***P value*** | **Differences [95% CI]** | ***P value*** | **Differences [95% CI]** | ***P value*** |
| MVPA (min/week) | 20 [4;36] | .02 | 21 [3;39] | .02 | 10 [-6;26] | .23 | 10 [-8;28] | .27 | 10 [-3;24] | .14 | 11 [-4;26] | .15 |
| Moderate intensity (min/week) | 5 [-3;13] | .22 | 6 [-4;16] | .23 | 3 [-5;11] | .45 | 4 [-6;13] | .47 | 2 [-5;9] | .57 | 2 [-6;10] | .58 |
| Vigorous intensity (min/week) | 13 [4;22] | .007 | 13 [3;22] | .009 | 4 [-5;13] | .39 | 3 [-6;13] | .47 | 9 [1;16] | .02 | 9 [1;17] | .02 |
| Steps (steps/day) | 251 [-173;674] | .24 | 136 [-274;546] | .51 | 149 [-272;571] | .49 | 32 [-375;440] | .88 | 102 [-246;449] | .57 | 104 [-233;441] | .54 |
| Active time (min/day) | 4 [-4;12] | .30 | 3 [-5;10] | .50 | 4 [-4;12] | .36 | 3 [-5;11] | .48 | 0.5 [-6;7] | .89 | 0.1 [-6;6] | .98 |
| Active kilocalories (kcal/day) | 25 [-15;64] | .22 | 15 [-32;62] | .52 | 29 [-10;69] | .15 | 30 [-17;77] | .20 | -5 [-37;28] | .78 | -15 [-53;24] | .44 |
| Floors climbed (floors/day) | 1 [-0.2;1] | .16 | -0.1 [-1;1] | .84 | 1 [-0.1;1] | .07 | -0.1 [-1;1] | .91 | -0.2 [-1;0.5] | .62 | -0.04 [-1;1] | .92 |
| Minimum heart rate (beats/min) | -0.5 [-1;1] | .26 | -0.3 [-1;1] | .62 | 0.1 [-1;1] | .86 | -0.4 [-1;1] | .42 | -1 [-1;0.1] | .12 | -1 [-2;0.1] | .11 |
| Maximum heart rate (beats/min) | 2 [0.3;3] | .02 | 1 [-0.4;3] | .14 | 1 [-1;2] | .27 | 0.3 [-1;2] | .68 | 1 [0;2] | .14 | 1 [-0.4;2] | .20 |
| Resting heart rate (beats/min) | -0.2 [-1;1] | .63 | -0.02 [-1;1] | .97 | 0.3 [-1;1] | .52 | 1 [-0.5;2] | .28 | -1 [-1;0.2] | .17 | -1 [-1;0.3] | .18 |
| Average heart rate (beats/min) | 0.03 [-1;1] | .94 | 0.02 [-1;1] | .97 | 0.5 [-0.4;1] | .26 | 1 [0.4;2] | .22 | -0.5 [-1;0.3] | .20 | -1 [-1;0.2] | .15 |

A positive mean value indicates that the last-mentioned group has the highest mean. MVPA, sum of moderate and vigorous intensity physical activity (PA) in min/week; moderate intensity PA, cumulative duration of activities of moderate-intensity (MET=3-6) lasting at least 10 consecutive min in min/week; vigorous intensity PA, cumulative duration of activities of vigorous-intensity (MET> 6) lasting at least 10 consecutive min in min/week; steps, steps counted per day; active time, active time in min/day; active kilocalories (Kcal), calories burned through actual movement in Kcal/day; floors climbed, number of floors climbed per day (a floor climbed is equal to 3 meters); minimum heart rate, the lowest heart rate in beats/min; maximum heart rate, the highest heart rate in beats/min; resting heart rate, the average of seven days of the resting heart rate in beats/min; average heart rate, the average heart rate in beats/min; visit 2, the 29^th^ gestational week.*Significant difference. CI, confidence interval; CON, standard care; EXE, structured supervised exercise training; MOT, motivational counseling on physical activity.
