# Supplementary figures and images for: Effects of Structured Supervised Exercise Training or Motivational Counseling on Pregnant Women’s Physical Activity Level: FitMum - Randomized Controlled Trial
Source: J Med Internet Res. 2022 Jul 20;24(7):e37699. doi: 10.2196/37699 (PMC9350815; doi:10.2196/37699)

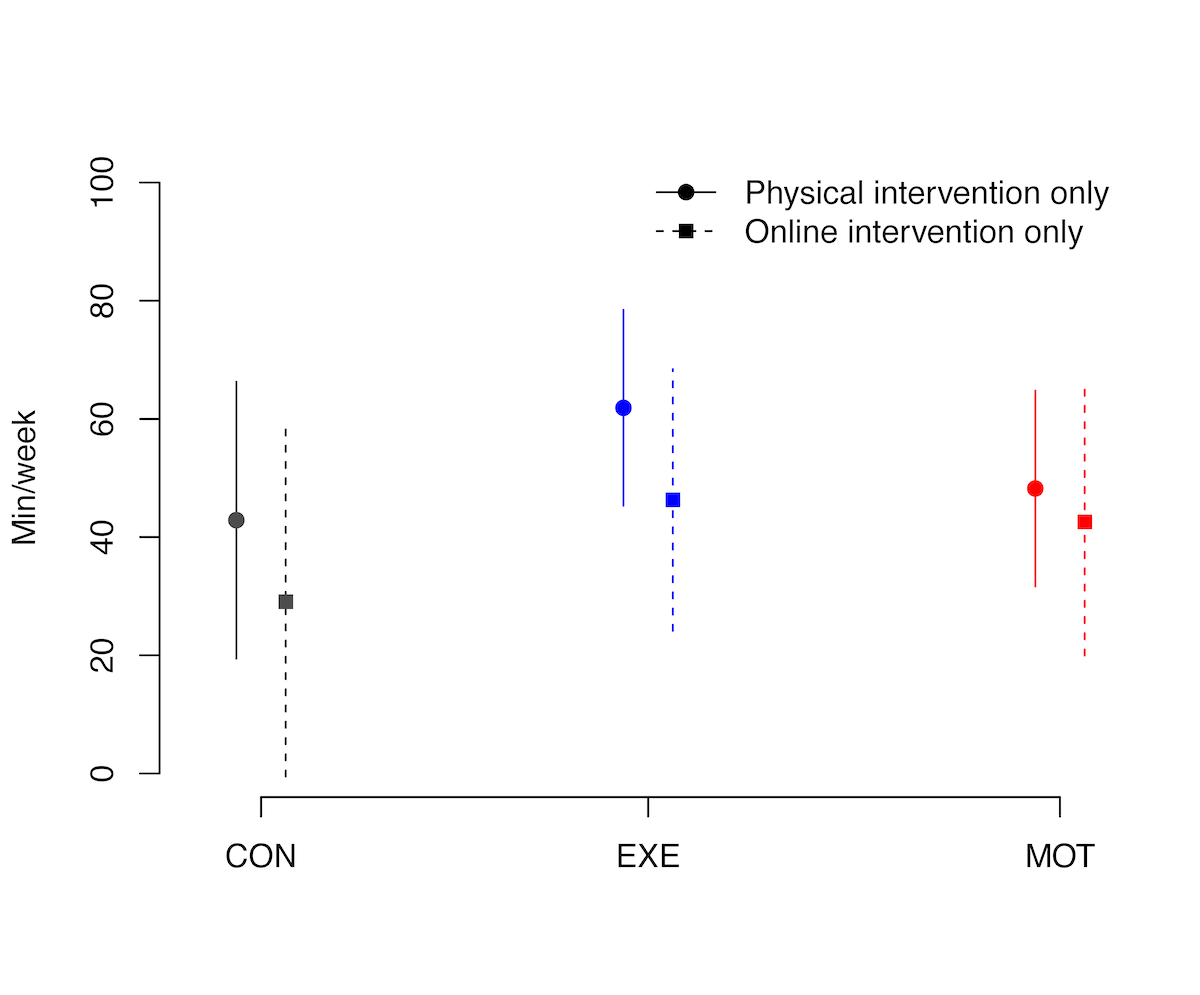

Supplement: Multimedia Appendix 2 [file jmir_v24i7e37699_app2.png]

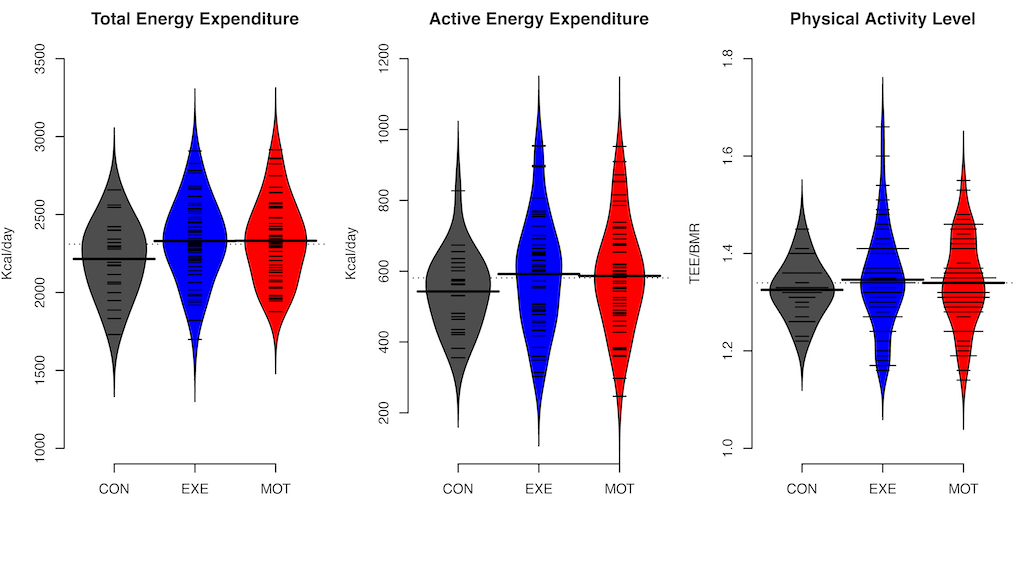

Supplement: Multimedia Appendix 5 [file jmir_v24i7e37699_app5.png]
