# Supplementary material for: Effects of Structured Supervised Exercise Training or Motivational Counseling on Pregnant Women’s Physical Activity Level: FitMum - Randomized Controlled Trial
Source: J Med Internet Res. 2022 Jul 20;24(7):e37699. doi: 10.2196/37699 (PMC9350815; doi:10.2196/37699)
Supplement: Multimedia Appendix 3 [file jmir_v24i7e37699_app3.docx]

Multimedia Appendix 3. Pregnancy Physical Activity Questionnaire outcome differences. Comparison between groups based on physical activity level from the Danish version of the Pregnancy Physical Activity Questionnaire (PPAQ-DK).

|  | CON vs EXE | | | | CON vs MOT | | | | MOT vs EXE | | | | |
| --- | --- | --- | --- | --- | --- | --- | --- | --- | --- | --- | --- | --- | --- |
|  | **Visit 2** | | **Visit 3** | | **Visit 2** | | **Visit 3** | | **Visit 2** | | **Visit 3** | |  |
|  | **Differences [95% CI]** | ***P value*** | **Differences [95% CI]** | ***P value*** | **Differences [95% CI]** | ***P value*** | **Differences [95% CI]** | ***P value*** | **Differences [95% CI]** | ***P value*** | **Differences [95% CI]** | ***P value*** |  |
| Total activity (MET-h/week) |  |  |  |  |  |  |  |  |  |  |  |  |  |
| Total activity | 3 [-13;19] | .70 | 12 [-8;32] | .23 | 4 [-12;20] | .61 | 2 [-18;22] | .85 | -1 [-14;12] | .88 | 10 [﻿-6;26] | .21 |  |
| Activity of ≥ light intensity | 6 [-10;23] | .45 | 12 [-7;31] | .23 | 6 [-11;22] | .51 | 1 [-19;21] | .94 | 1 [﻿-13;14] | .92 | 11 [﻿-4;27] | .16 |  |
| Intensity (MET-h/week) |  |  |  |  |  |  |  |  |  |  |  |  |  |
| Sedentary | -3 [-6;1] | .10 | -0.4 [-5;4] | .84 | -1 [-5;2] | .47 | 2 [-3;5] | .68 | -2 [-4;1] | .24 | -1 [-5;2] | .44 |  |
| Light | -2 [-13;10] | .80 | 6 [-17;12] | .23 | 2 [-9;14] | .69 | 6 [-19;10] | .54 | -4 [-14;6] | .41 | 2 [-10;13] | .74 |  |
| Moderate | 5 [-4;14] | .26 | 9 [-2;21] | .11 | 1 [-8;11] | .75 | 2 [-10;13] | .74 | 4 [-4;11] | .31 | 7 [-2;16] | .11 |  |
| Vigorous | 1 [﻿-1;3] | .23 | -0.2 [-2;2] | .82 | 2 [0;3] | .66 | 1 [﻿-1;3] | .41 | -1 [-2;1] | .41 | -1 [-2;0.4] | .18 |  |
| Type (MET-h/week) |  |  |  |  |  |  |  |  |  |  |  |  |  |
| Household | -1 [﻿-10;9] | .89 | 3 [﻿-9;14] | .65 | -4 [-13;6] | .43 | -1 [-13;11] | .83 | 3 [﻿-4;11] | .42 | 4 [-5;13] | .40 |  |
| Occupational | -0.4 [﻿-13;12] | .96 | -0.4 [-15;15] | .96 | 3 [-10;15] | .66 | -4 [-19;12] | .65 | -3 [-13;7] | .54 | 3 [﻿-9;15] | .60 |  |
| Sports | 6 [-1;6] | .001 | 6 [﻿2;10] | .003 | 3 [-1;6] | .12 | 2 [-2;6] | .40 | 3 [0.2;6] | .04 | 4 [﻿1;7] | .008 |  |
| Transportation | -1 [﻿-5;3] | .49 | 0.1 [﻿-4;4] | .95 | 1 [-3;5] | .73 | 1 [-3;5] | .73 | -2 [-5;1] | .19 | -1 [-4;2] | .72 |  |
| Inactivity | -2 [-6;2] | .26 | -0.1 [-5;5] | .98 | -1 [-4;3] | .71 | 2 [-3;6] | .54 | -1 [-4;2] | .35 | -2 [-5;2] | .41 |  |

A positive mean value indicates that the last-mentioned group has the highest mean. Visit 2, the 29^th^ gestational week; visit 3, the 29^th^ gestational week. CI, confidence interval; MET, metabolic equivalent of task; h/week, hours/week; CON, standard care; EXE, structured supervised exercise training; MOT, motivational counseling on physical activity.
