# Supplementary material for: Effects of Structured Supervised Exercise Training or Motivational Counseling on Pregnant Women’s Physical Activity Level: FitMum - Randomized Controlled Trial
Source: J Med Internet Res. 2022 Jul 20;24(7):e37699. doi: 10.2196/37699 (PMC9350815; doi:10.2196/37699)
Supplement: Multimedia Appendix 4 [file jmir_v24i7e37699_app4.docx]

Multimedia Appendix 4. Pregnancy Physical Activity Questionnaire outcome descriptive statistics.

|  | CON | | | | | EXE | | | | | MOT | | | | |
| --- | --- | --- | --- | --- | --- | --- | --- | --- | --- | --- | --- | --- | --- | --- | --- |
|  | **Visit 1** | **Visit 2** | **Visit 3** | **Time effect  visit 1 - visit 2 (*P*)** | **Time effect  visit 1 - visit 3 (*P*)** | **Visit 1** | **Visit 2** | **Visit 3** | **Time effect  visit 1 - visit 2 (*P*)** | **Time effect  visit 1 - visit 3 (*P*)** | **Visit 1** | **Visit 2** | **Visit 3** | **Time effect  visit 1 - visit 2 (*P*)** | **Time effect  visit 1 - visit 3 (*P*)** |
|  | **n=45** | **n=35** | **n=31** |  |  | **n=87** | **n=76** | **n=74** |  |  | **n=87** | **n=71** | **n=64** |  |  |
|  | Mean (SD) | | |  |  | Mean (SD) | | |  |  | Mean (SD) | | |  |  |
| Total activity (MET-h/week) |  |  |  |  |  |  |  |  |  |  |  |  |  |  |  |
| Total activity | 161 (43) | 154 (44) | 132 (52) | -7 (.34) | -29 (.001) | 150 (49) | 151 (50) | 136 (56) | 1 (.88) | -14 (.05) | 157 (52) | 155 (52) | 127 (45) | -2 (.83) | -30 (<.001) |
| Activity of ≥ light intensity | 147 (44) | 139 (44) | 117 (50) | -8 (.25) | -30 (<.001) | 135 (48) | 138 (49) | 121 (54) | 3 (.59) | -14 (.03) | 142 (50) | 140 (55) | 112 (47) | -2 (.85) | -30 (<.001) |
| Intensity (MET-h/week) |  |  |  |  |  |  |  |  |  |  |  |  |  |  |  |
| Sedentary | 13 (10) | 15 (13) | 15 (12) | 2 (.32) | 2 (.25) | 15 (12) | 12 (8) | 15 (12) | 3 (.07) | 0 (.31) | 14 (14) | 14 (14) | 16 (14) | 0 (.92) | 2 (.036) |
| Light | 112 (35) | 106 (34) | 91 (45) | -6 (.16) | -21 (<.001) | 98 (34) | 96 (33) | 81 (34) | -2 (.35) | -17 (<.001) | 108 (37) | 104 (41) | 82 (34) | -4 (.38) | -26 (<.001) |
| Moderate | 33 (21) | 32 (21) | 26 (20) | -1 (.91) | -7 (.30) | 36 (30) | 40 (29) | 37 (36) | 4 (.17) | 1 (.63) | 35 (27) | 35 (25) | 29 (22) | 0 (.74) | -6 (.21) |
| Vigorous | 1 (3) | 1 (2) | 2 (5) | 0 (.97) | 1 (.56) | 1 (3) | 2 (4) | 2 (3) | 1 (.09) | 1 (.89) | 1 (2) | 3 (5) | 2 (5) | 2 (.002) | 1 (.03) |
| Type (MET-h/week) |  |  |  |  |  |  |  |  |  |  |  |  |  |  |  |
| Household | 65 (36) | 63 (35) | 59 (31) | -2 (.84) | -6 (.42) | 54 (34) | 57 (39) | 56 (35) | 3 (.35) | 2 (.43) | 64 (44) | 60 (39) | 57 (36) | -4 (.36) | -7 (.18) |
| Occupational | 56 (29) | 49 (29) | 34 (32) | -7 (.13) | -22 (<.001) | 57 (37) | 47 (36) | 31 (42) | -10 (.02) | -26 (<.001) | 56 (32) | 50 (31) | 28 (30) | -6 (.14) | -28 (<.001) |
| Sports | 7 (7) | 9 (7) | 9 (10) | 2 (.06) | 2 (.14) | 7 (7) | 15 (11) | 15 (10) | 8 (<.001) | 8 (<.001) | 5 (5) | 11 (9) | 10 (10) | 6 (<.001) | 5 (<.001) |
| Transportation | 14 (9) | 14 (8) | 12 (10) | 0 (.79) | -2 (.32) | 13 (8) | 13 (13) | 12 (11) | 0 (.56) | -1 (.27) | 14 (9) | 15 (11) | 13 (9) | 1 (.37) | -1 (.34) |
| Inactivity | 15 (11) | 17 (13) | 18 (11) | 2 (.49) | 3 (.19) | 16 (13) | 15 (9) | 18 (12) | -1 (.27) | -2 (.10) | 16 (16) | 17 (14) | 19 (16) | 1 (.90) | 3 (.007) |

Unadjusted comparison of the raw mean ± SD and p-values from regression analysis within the groups, physical activity (PA) pattern and time effects from visit 1 to visit 2 and visit 3, respectively. Visit 1, gestational age of maximum 15 weeks and 0 days; visit 2, the 29^th^ gestational week; visit 3, the 35^th^ gestational week. SD, Standard deviation; MET, metabolic equivalent of task; h/week, hours/week; CON, standard care; EXE, structured supervised exercise training; MOT, motivational counseling on physical activity.
